# C57BL/6J mice as a polygenic developmental model of diet‐induced obesity

**DOI:** 10.14814/phy2.13093

**Published:** 2017-04-11

**Authors:** Dinh‐Toi Chu, Elzbieta Malinowska, Magdalena Jura, Leslie P. Kozak

**Affiliations:** ^1^ Institute of Animal Reproduction and Food ResearchPolish Academy of SciencesOlsztynPoland; ^2^ Present address: Centre for Molecular Medicine Norway (NCMM)Nordic EMBL PartnershipUniversity of Oslo and Oslo University HospitalNorway

**Keywords:** Developmental control of obesity, diet‐induced obesity, epigenetic mechanisms of obesity, genetic variation of obesity, lipogenic genes

## Abstract

Susceptibility to obesity changes during the course of life. We utilized the C57BL/6J (B6) and 129S mouse as a genetic model for variation in diet‐induced obesity to define the adiposity phenotypes from birth to maturity at 8 weeks‐of‐age. From birth to 8 weeks‐of‐age, both male and female 129S mice had significantly higher fat mass and adiposity index than B6 mice, although they were not obese. After 8 weeks‐of‐age, B6 had greater adiposity/obesity than 129S mice in response to a high fat (HF). We sought to determine the mechanism activating the fat accumulation in B6 mice at 8‐weeks‐of‐age. We used microarray analysis of gene expression during development of inguinal fat to show that molecular networks of lipogenesis were maximally expressed at 8 weeks‐of‐age. In addition, the DNA methylation analysis of the *Sfrp5* promoter and binding of acetylated histones to *Sfrp5* and *Acly* promoter regions showed that major differences in the expression of genes of lipogenesis and chromatin structure occur during development. Differences in lipogenesis networks could account for the strain‐dependent differences in adiposity up to 8 weeks‐of‐age; however, changes in the expression of genes in these networks were not associated with the susceptibility to DIO in B6 male mice beyond 8 weeks‐of‐age.

## Introduction

Obesity and its related disorders are complex quantitative traits determined by interactions between genetic variation in physiology and the environment. The genetic architecture of obesity, as analyzed by genome‐wide association studies (GWAS), has implicated over 50 individual genetic loci in determining obesity phenotypes (Rankinen et al. 2006). Genetic crosses, mutation, knockout, and transgenic animals have also been used as surrogate models of human obesity (Brockmann and Bevova 2002; Nilsson et al. 2012). Experimental animals of obesity can be classified into monogenetic and polygenetic models (Nilsson et al. 2012). The monogenetic laboratory animals of obesity are characterized by mutations in single genes having important roles on metabolism, for example, leptin gene in *ob/ob* (obese) mouse, *db/db* (diabetic) mouse, tub (tubby) mouse, Zucker fatty rats (ZFR), and carboxypeptidase E gene in *fat/fat* mouse (Brockmann and Bevova 2002). The polygenetic models of obesity (Brockmann and Bevova 2002) are lines of mice or other animals differing in body weight and fat deposition and they include inbred and recombinant inbred lines, chromosome substitution lines (Singer et al. 2004; Shao et al. 2008), the genetic collaborative cross (Chesler et al. 2008; Philip et al. 2011), and a number of traditional and advanced intercross lines (Darvasi and Soller 1995; Fawcett et al. 2008, 2010). The utility of these genetic tools to unravel the complexity of obesity, derived from variant alleles together with contributions from miRNAs and epigenetic modifications, have been reviewed (Pomp and Mohlke 2008; Pomp et al. 2008). However, the identification of genes and the mechanisms that explain the obesity phenotypes have not progressed to a real understanding of the functional contribution of the quantitative trait loci. A major problem for both humans and mice lies in being able to gauge the contribution of multiple genes each contributing in a modest way to the obesity phenotype.

One of the more intensively studied models of polygenic obesity has been the C57BL/6J and 129 inbred strains and their intercross progeny – (C57BL/6ByJx129P3/J) F2 (Reed et al. 2003, 2006; Lin et al. 2013). At least, 10 chromosomes (2, 4, 5, 7, 8, 9, 11, 12, 16, and 17) have loci implicated in the regulation of body weight, body fat, obesity, and the development of local fat depots (Reed et al. 2003, 2006; Lin et al. 2013). Of these, loci on chromosomes 2, 4, 9, and 16 determine body weight, body length, and adiposity (Reed et al. 2003). At the age of 6.8–12.6 months on a standard chow diet (STD), B6 mice are heavier and more obese than 129S mice with the more significant differences between strains being found in males (Reed et al. 2006). Dietary studies on adult male animals suggest that B6 mice consuming a high fat (HF) or a STD diet gained more weight with higher feeding efficiency than 129S mice (Almind and Kahn 2004; Almind et al. 2007). Developmental studies conducted during first 30 days of life, showed that 129S mice develop higher total fat mass with larger ING, EP, and RP fat depots than B6 mice. It has been proposed that this difference in obesity is due to differences in brown adipocytes and its effects on energy balance in weaning age mice (Lasar et al. 2013); however, this phenotype was not replicated in a more recent study from our laboratory (Chabowska‐Kita et al. 2015).

Elucidating the physiological/molecular basis of multifactorial obesity or diabetes is an incredibly difficult task to solve. One is dealing with a biological phenotype in which allelic variation at a single gene may contribute only one or two percent of the variance. Mutating these single genes in a mouse model can provide some insight, but not enough to provide significant mechanistic understanding. During the course of our studies on the basis of variation in diet‐induced obesity, it became evident that the variation in susceptibility to obesity was a consequence of the environment during the lactation period. Furthermore, it is clear from many studies that age‐dependent adiposity phenotypes result from the interaction of genetic and development factors (Frayling et al. 2007; Scuteri et al. 2007; Day and Loos 2011; Lu and Loos 2013; Loos and Yeo 2014). In recent studies, we tested the effects of changes to single genes, such as *Ucp1*,* Sfrp5*, and *Mest* as well as manipulation of diet and ambient temperature (Koza et al. 2006; Nikonova et al. 2008; Kozak et al. 2010; Chu et al. 2014; Chabowska‐Kita et al. 2015). What became clear was that the adipobiology of the lactating mouse is different than that of the young adult mouse, and that of the aging mouse may have its own unique characteristics (Chu et al. 2014). Given that the final phenotype is based upon an age‐dependent difference in adipose tissue expansion during diet‐induced obesity between B6 and 129 mice, differences in the metabolic pathways associated with lipid deposition must be involved. Although this difference may depend upon transcription factors, nevertheless, our hypothesis is that differences in the pathways of lipid deposition are ultimately necessary to realize the phenotypes. Accordingly, this paper describes a three pronged strategy; first, we established more rigorously the dependence/interactions of developmental age, diet, and genotype on adiposity and glucose metabolism phenotypes, and then we characterized gene pathways associated with lipid deposition and diet‐induced obesity from three sources, (1) those genes revealed from comprehensive gene expression studies of DIO in adult male B6 mice (Koza et al. 2006), (2) those genes that were induced in subcutaneous fat depots, irrespective of diet, between 10 days and 8 weeks‐of‐age (Kozak et al. 2010), and (3) those genes associated with increased obesity in QTL studies between B6 and 129 mice (Lin et al. 2013). Finally, we looked for changes in chromatin structure of two key markers of lipid metabolism to acquire insights into possible molecular mechanisms.

## Materials and Methods

### Mice

Breeding colonies of C57BL/6J and 129S1/SvImJ mice were generated with breeding pairs purchased from the Jackson Laboratory (Bar Harbor, Maine). Mice were conventionally housed at 23°C with a 12 h light/dark cycle. Breeders were fed a chow diet with 25% of calories from fat (Lab‐Diet 5015). Diets for postweaning mice were either a high fat diet (HF) with 59% of calories from fat (Test‐Diet, #9G03) or a STD with 13% of calories from fat (PicoLab Diet 20). Male mice were used in this study. All experimental protocols, conducted at the Institute of Animal Reproduction and Food Science, Polish Academy of Sciences, Olsztyn, Poland, were approved by the Local Committee for the Ethical Treatment of Experimental Animals of Warmia‐Mazury University (NR 38/2011) following guidelines of EU Directive 2010/63/EU and Pennington Biomedical Research Center's Institutional Animal Care and Use Committee.

### Animal protocol

Because susceptibility to DIO in B6 mice is highly influenced by nutritional status during the lactation period (Kozak et al. 2010), we tested how susceptibility to DIO of B6 and 129 pups and adult offspring could be influenced by the maternal environment by cross‐fostering B6 pups to the 129 mother and 129 pups to the B6 mother (experiment conducted at the Pennington Biomedical Research Center). Within a day of birth, pups were sorted to provide eight pups per dam and maintained with the dam‐fed Lab‐Diet 5015 until they were weaned at 21 days of age at four pups per pen (females and males were not separated) and fed PicoLab Diet 20. At 7 weeks‐of‐age, they were individually housed for 1 week; at 8 weeks‐of‐age, they were fed a high fat diet for 8 weeks. Based upon the results of the cross‐fostering experiment, subsequent studies were conducted with 3 or 8‐week‐old mice of both strains fed with a STD or HF at room temperature until they were 19 or 31 weeks‐of‐age (experiment conducted in Poland). Body composition was analyzed by an NMR analyzer (Minispec LF90II; Bruker) at 3, 8, 12, 16, 19, 26, and 31 weeks‐of‐age. Body weight (BW) was also measured at these time points. At the end of protocols (19 or 31 weeks‐of‐age), mice were killed to collect plasma, adipose tissues, and livers. Samples were snap frozen in liquid nitrogen and stored at −80°C until further analysis.

### Glucose and insulin tolerance tests

Glucose tolerance and insulin tolerance tests were performed as described previously (Anunciado‐Koza et al. 2008)***.*** For the glucose tolerance test (GTT), after fasting for 4 h from 8:00 to 12:00, mice received an intraperitoneal injection of 20% glucose solution (2 g/kg of body weight; Sigma G7528). For the insulin tolerance test (ITT), after fasting for 4 h from 8:00 to 12:00, mice received an intraperitoneal injection of insulin (0.75 IU/kg of body weight; Caninsulin, Intervet #256A01). Blood glucose levels were determined using a blood glucose monitor (ACCU‐CHEK^®^).

### RNA isolation

Total RNA from ING, RP, EP, and iBAT tissue was isolated by Tri‐reagent (Molecular Research Center, TR118) as described (Rim et al. 2004; Nikonova et al. 2008; Chu et al. 2014; Jura et al. 2015). RNA concentration was determined with a NanoDrop spectrometer. In some independent experiments, total RNA isolated from samples using RNeasy Mini Kit (Qiagen, 74106) gave similar results.

### qRT‐PCR analysis

qRT‐PCR was performed as described (Rim et al. 2004; Nikonova et al. 2008; Chu et al. 2014; Jura et al. 2015). TaqMan probes (5′‐6FAM and 3′‐TAMRA for each probe) were used for quantification of target genes using TaqMan^®^ One‐Step RT‐PCR Master Mix Reagents Kits (Life Technologies, 4313803). Sequences of primers and Taqman probes for targeted genes are taken from published papers (Rim et al. 2004; Si et al. 2007; Nikonova et al. 2008) and are available upon request. Gene expression data of target genes was calculated per 60 ng input RNA and normalized to cyclophilin (Cyclo).

### Western blot analysis

Frozen tissues were homogenized with an ice‐cold RIPA buffer. RIPA buffer contained 50 mmol/L Tris‐HCl pH 7.4, 150 mmol/L NaCl, 1 mmol/L EDTA, 0.1% SDS, 1% NP40, 0.25% sodium azide, with 10 *μ*L protease inhibitor cocktail (Sigma, P8340), 1 mmol/L phenylmethylsulfonyl fluoride (PMSF–Sigma, P7626), and 1 mL PhosStop solution/10 mL RIPA (PhosStop – Roche, 4906837001). Protein concentration was measured with the Bradford reagent (Sigma, B6916). Western blot analysis was performed as described (Rim et al. 2005; Anunciado‐Koza et al. 2008; Chu et al. 2014). The blots were incubated with antibodies against SCL25A1 (rabbit anti‐Scl25a1 – Santa Cruz Biotech sc‐86392) (1:200), ACLY (Rabbit anti‐Acly, Abcam, ab40793) (1/2000), MEST (Rabbit anti‐MEST (Nikonova et al. 2008)) (1:700), and *β*‐actin (mouse anti‐*β*‐actin – Abcam, Ab6276) (1:10000). Specific antibody–antigen complexes were detected using fluorescent‐labeled secondary antibodies (goat anti‐rabbit IRDye 800, Rockland, 611‐132‐122; goat anti‐mouse IRDye 800, Rockland, 610‐730‐124; Donkey anti‐goat IRDye 700, Licor, 926‐32214 and Donkey anti‐mouse IRDye 700, Rockland, 610‐730‐124). Bands were visualized and quantified using the Odyssey imaging system (Licor Bioscience). *β*‐actin was used as an internal control to adjust for variability in protein loading and transfer.

### Microarray analysis

For this experiment, we used data from a previous analysis of mouse inguinal fat as a function of developmental age and diet with the Applied Biosystems Mouse Genome Survey Microarray (Kozak et al. 2010). Each microarray contained approximately 34,000 features with a set of about 1000 controls. Signal intensities across microarrays were normalized using the quantile–quantile method (www.bioconductor.org). Microarray experiments, described according to MIAME guidelines, have been deposited in the GEO repository with the accession number GSE 19809.

### Bioinformatic analysis

#### Selection of potential genes

Genes involved in fat expansion and DIO during development were selected using microarray data from a previous study on development of ING fat in B6 male mice at 5, 10, 21, 56, and 112 days of age fed a standard diet (Kozak et al. 2010). Briefly, 328 genes upregulated at Day 56 versus Day 5, 10, and 21 were selected based on the ratio values of 5, 10, and 21 days to 56 days. Then, this list of upregulated genes was shortened using the Acumenta Literature Lab Viewer (http://www.acumenta.com/acumenta/index.php) to select 22 genes strongly related to lipid metabolism. The chromosome positions of these genes were identified by http://www.informatics.jax.org/javawi2/servlet/WIFetch?page=markerQF, and then matched to chromosomes in papers of Reed and colleagues (Reed et al. 2003, 2006; Lin et al. 2013). Percentages of selected genes related to fatty acid biosynthesis, oxidation of polyunsaturated fatty acid, and fatty acid metabolism were also determined by Acumenta Literature Lab Viewer. Finally, we selected six candidates (*Acss2, Acly, Elvol6, Scd1, Acaca,* and *Slc25a1*) located in chromosomal regions having an obesity‐related QTLs and a high probability of involvement in lipid homeostasis (Table 1).

**Table 1:** Candidate genes of lipogenesis selected from QTL analysis

Gene symbol	Gene name	Matching chromosome position		Percentage relevant to lipid homeostasis (By acumenta literature lab viewer)		
		Chr of our genes	Chr in Reed's papers (Reed et al. 2003, 2006; Lin et al. 2013)	Fatty acid biosynthesis (%)	Oxidation of polyunsaturated fatty acid (%)	Fatty acid metabolism (%)
Acss2	Acyl‐CoA synthetase short‐chain family member 2	Chr 2 77.26 cM	D2Mit224 74.0	0.86	0.08	1.00
Acly	ATP citrate lyase	Chr 11 63.47 cM	D11Mit199 62	24.65	0.08	0.08
Elovl6	ELOVL family member 6, elongation of long‐chain fatty acids (yeast)	Chr 3 58.05 cM	D3Mit10 49.7	1.76	0.56	7.04
Scd1	Stearoyl‐coenzyme A desaturase 1	Chr 19 37.98 cM	D19Mit11 41	19.09	13.66	56.64
Acaca	Acetyl‐coenzyme A carboxylase alpha	Chr 11 51.29 cM	D11Mit41 49	50.96	0.88	17.76
Slc25a1	Solute carrier family 25 (mitochondrial carrier, citrate transporter), member 1	Chr 16 11.11 cM	D16Mit3 21	0.02	—	—

(Chr, chromosome; D4Mit42, DNA segment, Chr 4, Massachusetts Institute of Technology 42).

#### Ingenuity pathway analysis

Microarray data from our previous study (Kozak et al. 2010) was employed to select pathways of metabolism induced during development between 10 and 56 days of age. Briefly, 374 upregulated and 431 downregulated genes at D56 versus D10 in three diet conditions (control, overnutrition, and undernutrition) were analyzed online using Ingenuity Pathway Analysis (IPA^®^, QIAGEN Redwood City, www.qiagen.com/ingenuity). Eighteen networks upregulated and 22 networks downregulated in ING fat of D56 compared to D10 B6 mice were identified (see Fig. 3).

### DNA isolation and methylation analysis

DNA was extracted from stromal vascular (SVF) and mature adipocyte fraction (MAF) obtained from ING of B6 males by the phenol–chloroform–isoamylalcohol (25:24:1, v/v) (Sigma, 77617) and EtOH precipitation. DNA pellet was dissolved in 50 *μ*L of 0.1x TE buffer. Bisulfite conversion of 1 *μ*g genomic DNA was performed with an EZ DNA Methylation^™^ kit according to manufacturer's instruction (Zymo Research, D5001). Bisulfite‐converted DNA was eluted in 10 *μ*L of 10 mol/L Tris‐EDTA buffer. The target region of Sfrp5 was amplified by PCR with the following primers: forward 5′‐gTtgTagattggTtagggaa‐3′; and reverse 5′‐ ActtAcctActActtcacct‐3′. Cloning of bisulfite‐converted DNA was done using the TOPO^®^ TA Cloning Kit for Sequencing (Life Technologies, # K4575‐01). PCR amplicons of Sfrp5 was ligated into pCR^™^4‐TOPO^®^ vector in the presence of salt (200 mmol/L NaCl, 10 mmol/L MgCl_2_). Transformation was carried out according to manufacturer's instructions. Only bacteria with ligated amplicons grew on the selective LB plates. Ten individual clones for each cellular fraction and each experiment were picked for PCR amplification with M13 primers, then sequenced and analyzed. DNA Sequencing was performed by a fee‐for‐service company (Centrum Badań DNA, Poznań, Poland). A web‐based tool was used for bisulfite sequence analysis (NCBI/BLAST tools). The significance of the DNA methylation data (Fig. 6F) was determined by the Student's *t*‐test to compare the mean percentage values of methylation of the 46 CpG's islands in the promoter region of *Sfrp5* gene in the MAF and the SVF from ING fat tissue with *n* = 30, 40, and 40 clones for 10 days, 8 weeks, and 12 weeks‐of‐age mice, respectively.

### Chromatin isolation and chromatin immunoprecipitation assay (ChiP assay)

Chromatin was isolated from frozen or fresh adipose tissues collected from adult C57BL/6J or 129S males by steps involving crosslinking, stopping crosslinking and DNA shearing as described by previous articles (Neess et al. 2011; Haim et al. 2013). Sheared chromatin between 100 and 1000 bp was diluted 1:10 with cold IP dilution buffer (0.01% SDS, 1.1% Triton X‐100, 1.2 mmol/L EDTA, 16.7 mmol/L Tris‐HCl, 167 mmol/L NaCl), then incubated with 5–10 *μ*L of primary antibodies of interest (Anti‐acetyl‐Histone H3‐Rabbit antibody, Millipore, Cat. #06‐599; Anti‐Histone H4 antibody – Rabbit, Abcam, ab15823; or Anti‐Acetyl ‐Histone H2B antibody – Rabbit, Abcam, ab1759) with rotation overnight at 4°C. Then, 5 *μ*L of the biotinylated secondary antibody (Donkey anti‐rabbit IgG H&L, Abcam 7082) was added, and incubated for 1–2 h at 4°C on a rotating device. Negative controls were incubated without addition of antibodies. A total quantity of 25 *μ*L of washed streptavidin magnetic beads (88817, Thermo Scientific) was added to each sample followed by incubation at 4°C for 1 h. Pellet magnetic beads were collected and sequentially washed once with low salt buffer (0.1% SDS, 1% Triton X‐100, 2 mmol/L EDTA, 20 mmol/L Tris‐HCl, pH 8.1, 150 mmol/L NaCl), high salt buffer (0.1% SDS, 1% Triton X‐100, 2 mmol/L EDTA, 20 mmol/L Tris‐HCl, pH 8.1, 500 mmol/L NaCl), LiCl buffer (250 mmol/L LiCl, 1% NP‐40, 1% Na‐deoxycholate, 1 mmol/L EDTA, 10 mmol/L Tris, pH 8.1), and TE buffer (10  mmol/L Tris‐HCl pH 8.1, 1 mmol/L EDTA)(Nie et al. 2009). The DNA‐chromatin‐antibodies‐complex was eluted from beads by adding 200 *μ*L IP elution buffer (1% SDS, 100 mM NaHCO3) and incubating at RT for 15 min with rotation. DNA was purified by the standard phenol/chloroform extraction method after decrosslinking, and amplified by qPCR in duplicate or triplicate using a 2X SYBR green reaction mix (Quanta biosciences, 95054‐100) on 384‐well reaction plates (Life Technologies). Sequences of primers for adipose biofunctional markers (Acly, Scl25a1, and Sfrp5) were taken from published studies (Linhart et al. 2007; Neess et al. 2011; Damiano et al. 2012). PCRs were run on a 7900HT sequence detection system (Applied Biosystems) and the cycle threshold (*C*_t_) values from the qPCR assay were collected for analysis.

### Statistical analysis

Data are expressed as mean ± SD or mean ± SEM. Analyses were performed using GraphPad Prism 5.0. Student's *t*‐test was used for single comparisons and one‐way or two‐way ANOVA for multiple group comparisons. Unless otherwise specified, *,+,$ *P *≤ 0.05; **,++, *P* ≤ 0.01 and ***,+++,$ *P* ≤ 0.001; and not significant (n.s) *P* > 0.05.

## Results

### Effects of developmental age and diet on phenotypic variation in body composition between B6 and 129 mice in a cross‐fostering design

**Figure 1:**
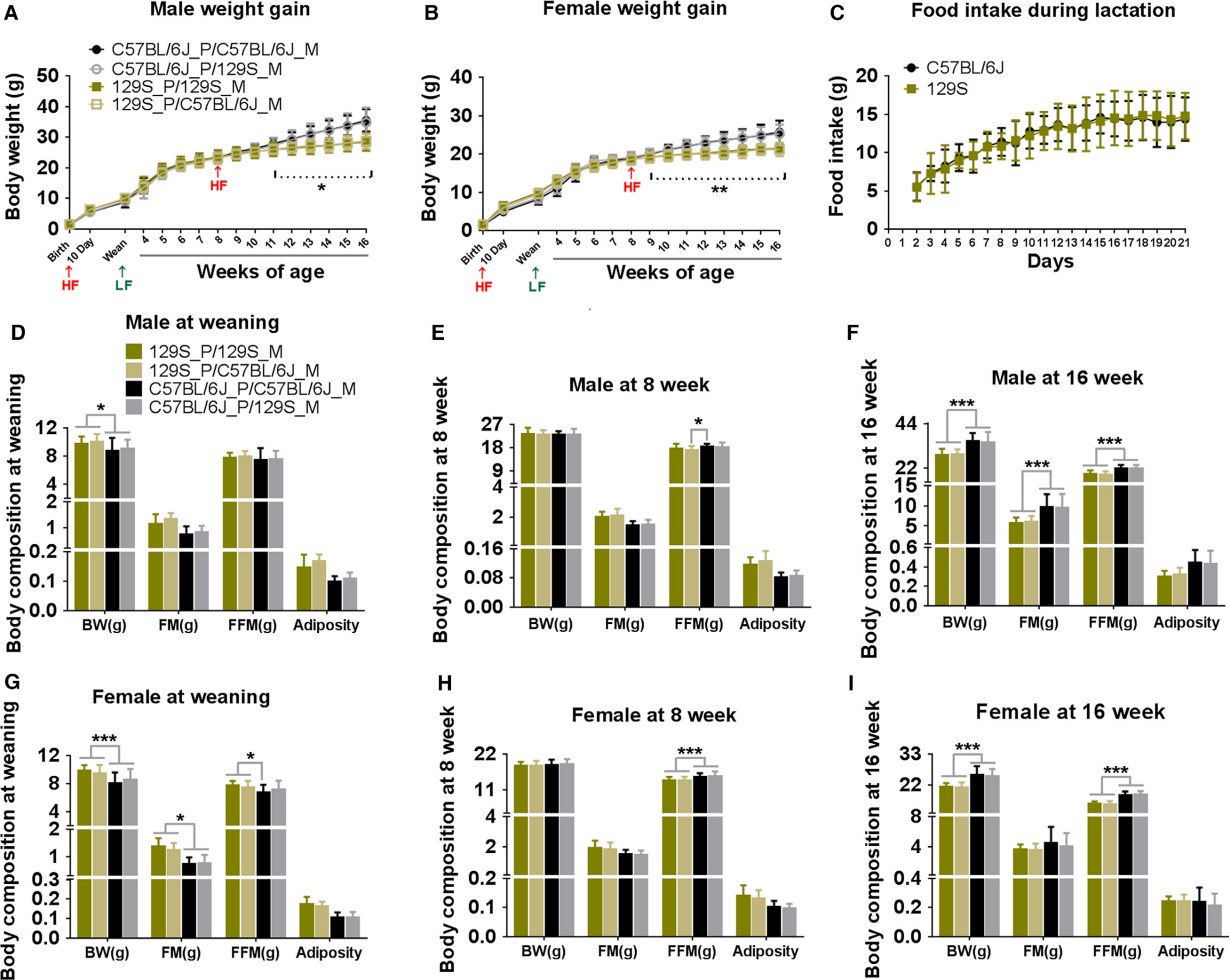
Cross‐fostering protocol indicates that variations in adiposity in B6 and 129 mice fed a HFD are independent of the genotype of nursing mother. C57BL/6J (B6) and 129S (P) litters were nursed by either B6 or 129S mothers fed a HF. After weaning, pubs were fed a low fat diet (LF) until 56 days of age, and then changed to a high fat to the end of experiment (16 weeks‐of‐age). Body weight of male pups (A) and female pups (B) were measured at indicated time points as shown in graphs, *n* = 18–37. Food intake of C57BL/6J and 129S dams was measured daily during lactation (C), *n* = 15. Body composition of male and female pups was determined at 3 weeks (D and G), 8 weeks (E and H), and 16 weeks‐of‐age (F and I)), *n* = 18–37. Data shown in graphs are the mean ± SD (error bars). Significance levels, indicated by (*), are for B6 mice nursed with either the B6 or 129S dam compared to 129S offspring nursed with either dam. Differences between groups were analyzed for statistical significance by two‐way ANOVA with multiple comparisons, significance levels indicated by *P ≤ 0.05, **P ≤ 0.01, and ***P ≤ 0.001. This experiment was performed at the Pennington Biomedical Research Center in Baton Rouge Louisiana; all other experiments were performed in Poland.

**Figure 2:**
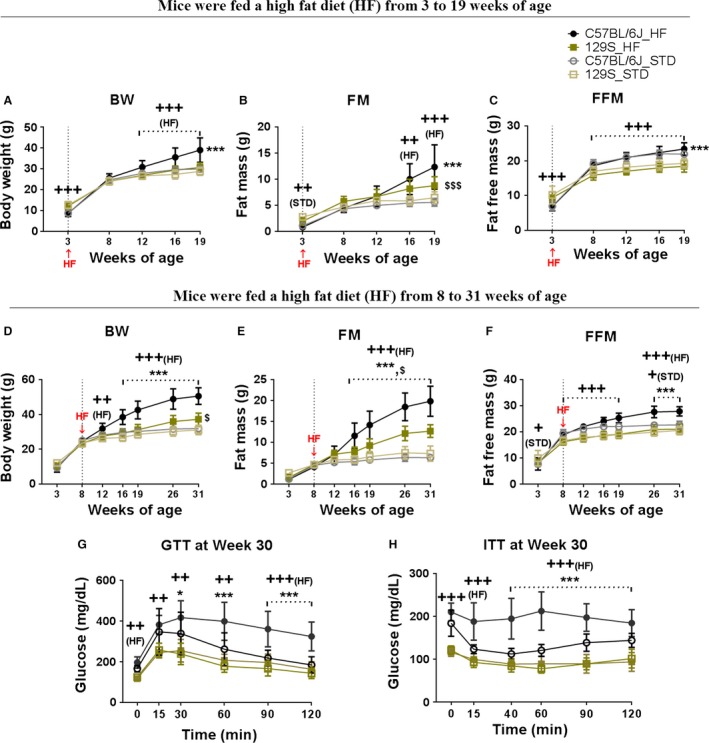
Variation in DIO between C57BL/6J and 129S mice initially consuming high fat diet from 3 or 8 weeks‐of‐age. C57BL/6J (B6) and 129S males were fed with a high fat diet (HF) or a STD from 3 weeks until 19 weeks‐of‐age, and from 8 weeks until 31 weeks‐of‐age. Body weight (A and D), fat mass (B and E), and fat‐free mass (C and F) were measured at indicated time points (3, 8, 12, 16, 19, 26, and 31 weeks‐of‐age). At 30 weeks‐of‐age, glucose tolerance (G) and insulin sensitivity (H) were tested in all groups. Data shown in graphs are the mean ± SD (error bars) with *n* = 11–16 (A–C) and 8–11 (D–H). Significant levels are indicated by (*) B6 fed with a HF (C57BL/6J _HF) group relative to B6 fed with a STD (C57BL/6J_STD) group, (^$^) 129S fed with a HF (129S_HF) group relative to 129S fed with a STD (129S_STD) group, and (^+^) B6 group relative to 129S group in the same diet. Differences between groups were analyzed for statistical significance by two‐way ANOVA with multiple comparisons, significance levels indicated by *,^+^,^S^P≤0.05, **,^++^,^SS^ *P* ≤ 0.01, and ***,^+++^,^SSS^ *P* ≤ 0.001. STD, standard chow diet

Adult male B6 fed a high fat diet are susceptible to the development of obesity, whereas under similar dietary conditions, 129 mice are resistant to the obese phenotype (Almind and Kahn 2004; Almind et al. 2007). Since the maternal milk of mice has a high fat content (fat content of 29.4% weight/volume) (Görs et al. 2009), we determined whether the high fat content of maternal milk would lead to greater adiposity in B6 than 129 pups from birth to weaning and from 3 to 8 weeks‐of‐age. Differences in the quantity and/or quality of maternal milk or even differences in maternal grooming and stress‐related behaviors may influence susceptibility to DIO in developing B6 and 129 mice (Meaney and Szyf 2005). We therefore cross‐fostered B6 and 129 pups from birth to weaning and assessed the development of adiposity during the preweaning period and then in mice fed a high fat diet (59% of calories from fat; TestDiet, #9G03) from 8 to 16 weeks‐of‐age. The profile of weight gain, adiposity, and food intake from birth to 16 weeks‐of‐age for both B6 and 129 males and females, nurtured by its birth‐mother or cross‐fostered, is shown Figure 1. The salient features are:

1. Viewed from birth to 16 weeks‐of‐age the most striking difference was an increase in body weight and adiposity in male B6 mice following the feeding of a high fat diet between 8 and 16 weeks‐of‐age (Fig. 1A and B). Even though from birth to approximately 15 days of age, the milk provided to the pups is a high fat diet, this did not lead to significantly higher adiposity in male B6 pups, in fact adiposity was higher in 129 male and female pups (Fig. 1D and G) (Kozak et al. 2010). Food intake by C57BL/6J and 129S dams was indistinguishable during the lactation period from birth to 21 days of age (Fig. 1C). Unexpectedly, fat mass and adiposity from birth to 8 weeks‐of‐age continued to be higher in 129S mice (Fig. 1D, E, G, and H), but was reversed following the feeding of a high fat diet from 8 to 16 weeks‐of‐age (Fig. 1F and I).
2. Cross‐fostering from birth to weaning did not change the development of body composition in B6 or 129 offspring (Fig. 1D and G), that is, body composition of male and female pups was determined by the genotype of the pup and not the nursing mother (Fig. 1D and G). Furthermore, the maturation period from weaning to 8 weeks‐of‐age, and diet‐induced adiposity in B6 or 129S mice that developed between 8 to 16 weeks‐of‐age was not affected by the genotype of the nursing mother (Fig. 1F).
3. 129S mice had more fat mass than B6 mice at weaning (already evident at D10, data not shown) and in 8 week‐old‐mice, thereby resulting in a higher adiposity index (Fig. 1D, E, G, and H), since fat‐free mass was not different between strains up to 8 weeks‐of‐age. These differences affected both males and females equally. Similar differences in preweaning mice have also been reported by Lasar et al. (2013).
4. At 8 weeks‐of‐age, mice were fed a high fat diet. After 8 weeks, male B6 mice had increased fat mass, lean mass, and adiposity index (Fig. 1F). Female mice did not increase fat mass when fed an obesogenic high fat diet, but they did increase fat‐free mass (Fig. 1I). Male and female B6 mice tended to have slightly higher fat‐free mass than 129S mice at both 8 and 16 weeks‐of‐age (Fig. 1H and I).
5. The increased sensitivity to DIO by B6 mice was not observed until the males were fed a high fat diet at 8 weeks‐of‐age. Female B6 and both male and female 129S mice were resistant to DIO (Fig. 1F and I); their adiposity indices did not exceed 0.2. At 16 weeks‐of‐age, after 8 weeks on HF, the indexes of B6 and 129S male mice were approximately 0.4 and 0.3, respectively. In our laboratory, using NMR analysis to determine body composition, only the B6 males are considered as obese (Fig. 2B and E). Consistent with the data on adiposity, long‐term feeding of HF caused glucose and insulin intolerance only in B6 males, not 129S males (Fig. 2G and H). Although the 129 females and males had higher fat mass at weaning and at 8 weeks‐of‐age, their adiposity index did not exceed 0.2 (Fig. 1D, E, G and H).

In summary, the cross‐fostering experiment has shown that between birth and 8 weeks‐of‐age, both male and female 129 mice have significantly higher fat mass and adiposity index than B6 mice; neither the genotype of the nursing dam nor the level of food intake by the dam affects the adiposity phenotype of the B6 or 129S strains. After 8 weeks‐of‐age, both male and female B6 have greater adiposity in response to a HF, but only male B6 mice had a level of adiposity (>0.4) indicative of obesity and only the male B6 mice were glucose intolerant and insulin resistant.

### The age at which B6 mice are more sensitive to diet‐induced obesity and diabetes

To assess the age when mice become sensitive to induction of obesity by HF, a new experiment was conducted in Poland in which cohorts of B6 and 129S male mice were fed a high fat and STD diets beginning at 3 weeks‐of‐age (Fig. 2A–C) and at 8 weeks‐of‐age (Fig. 2D–F). When the HF was begun at 8 weeks‐of‐age accelerated adiposity occurred in male B6, whereas the adiposity in 129S mice was attenuated (Fig. 2D–F); this was very similar to the observations at Pennington from 2009 to 2010 (Fig. 1D–F). However, when we began to feed the HF at 3 weeks‐of‐age, an obese state did not develop in the B6 mice until after 8 weeks‐of‐age (Fig. 2A–C). The time course of development of body composition of mice fed a HF at 3 weeks‐of‐age could be imposed on that of animals fed a HF beginning at 8 weeks‐of‐age. Accordingly, male B6 mice, similar to female B6 and male and female 129S mice, are resistant to DIO until they are 8 weeks‐of‐age. Furthermore, this sensitivity to DIO is observed irrespective of the genotype of the nursing dam. Only male B6 mice are sensitive to DIO and the phenotype is similar with either a B6 or 129 nursing mother.

Consistent with the adiposity phenotypes, at 30 weeks‐of‐age, B6 mice fed the HF were hyperglycemic under fasted conditions, glucose intolerant, and insulin resistant (Fig. 2G and H); however, it is evident that B6 mice fed a STD also showed signs of fasted hyperglycemia. It is noteworthy that there were no differences in glucose and insulin responses of 129S males fed a HF vs. STD (Fig. 2G and H), despite the fact that 129S males on a HF had quite a high fat mass (Fig. 2B and E). When the adiposity data from Figures 1 and 2 are considered together, we conclude that DIO in mice fed a HF occurs in B6 mice and not in 129S mice and that male B6 mice are not sensitive to DIO until they are 8 weeks‐of‐age.

### Candidate genes associated with metabolic networks contribute to differences in sensitivity to DIO in B6 and 129S mice during development

#### Microarray analysis of gene expression

We aimed to identify molecular mechanisms that could support the excessive accumulation of fat stores in B6 male mice at 8 weeks‐of‐age, but not at 3 weeks‐of‐age or earlier. We reasoned that a change in sensitivity to obesity in response to being fed a HF occurs shortly after 8 weeks‐of‐age. Histologically, the adipocytes in the inguinal fat depot of a 10‐day‐old mice are as well‐developed morphologically as an 8‐week‐old mice (Nikonova et al. 2008; Kozak et al. 2010), yet at 8 weeks‐of‐age, the mouse becomes obese when fed a high fat diet, whereas at 10 days of age or 3 weeks, obesity does not develop. An age‐dependent response is different in these mice existing in an obesogenic environment. To identify these differences, we mined microarray data from earlier developmental studies of gene expression in ING fat of B6 mice from the perspective that a 10‐day‐old mouse does not develop obesity when fed a HF, whereas an 8‐week‐old mouse will (Kozak et al. 2010). We assumed that metabolism associated with the accumulation of lipid was different between the male B6 at 10 versus 56 days of age and that the variation was genetically constitutive, and therefore present under the three nutritional conditions (control, C, overnutrition, O, and undernutrition, U). The design for analysis of the microarray data from Kozak et al. 2010 was to select genes in which increased expression between 10 and 56 days of age occurred irrespective of the nutritional environment.

**Figure 3:**
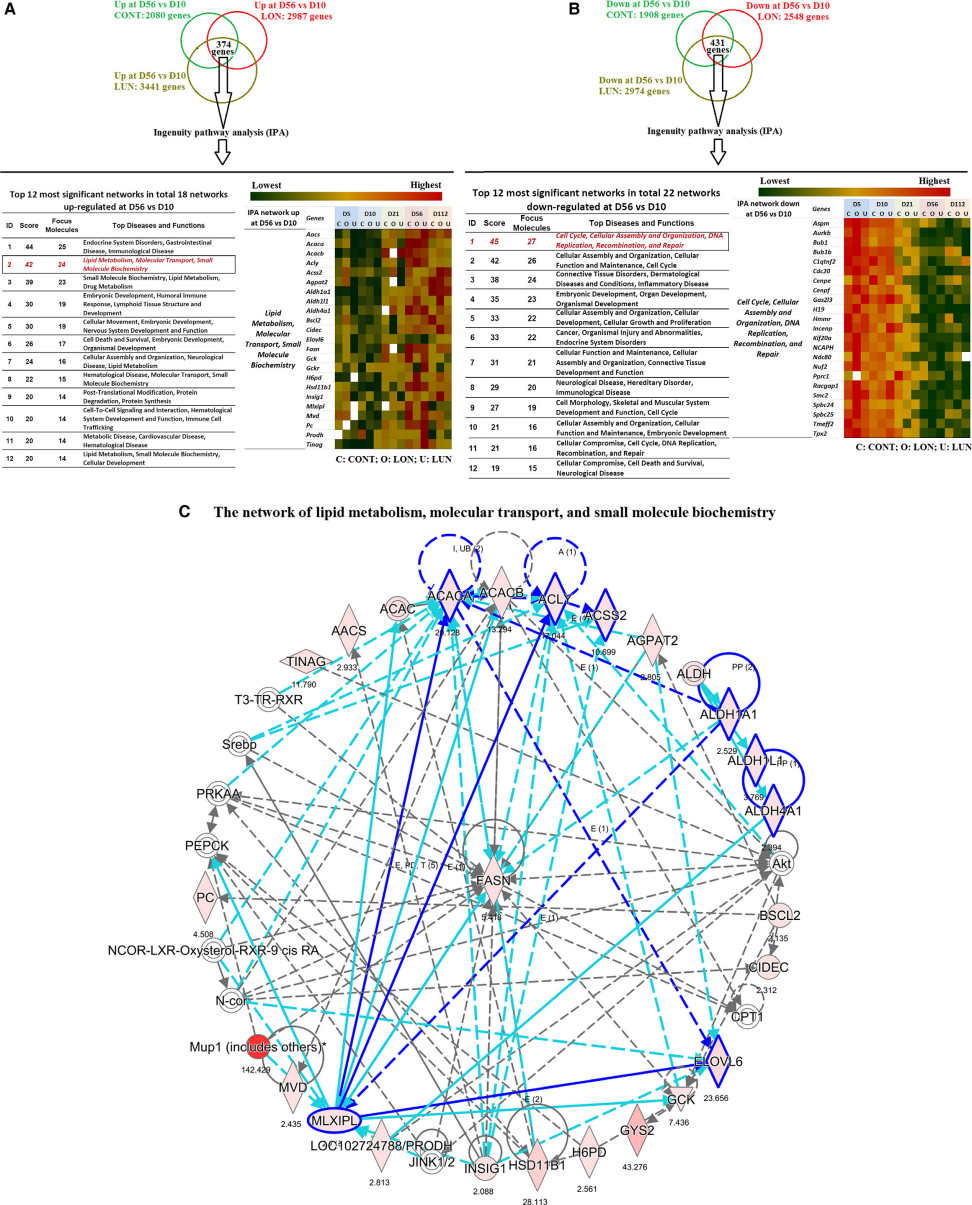
Up‐ and downregulated networks of development and metabolism in inguinal fat of C57BL/6J mice at 56 days (D56) versus 10 days (D10) of age. Microarray gene expression data was obtained from ING fat tissues of C57BL/6J mice at 5, 10, 21, 56, and 112 days of age. From birth to weaning (21 days of age), mice were kept with mothers who were fed a standard diet (Control – CONT), or a HF (overnutrition – LON), or a STD with reduced food availability (undernutrition – LUN) as described in Kozak et al. (Kozak et al. 2010). From 21 to 56 days of age, all offspring were fed a STD, then from 56 to 112 days of age, they were fed a HF. Fifteen experimental groups (3 diets × 5 ages) each contained a pooled of RNA from 12 mice. Each pool of RNA was analyzed by microarrays in triplicate. (A) Upregulated networks were obtained using IPA ^®^, QIAGEN Redwood City, www.qiagen.com/ingenuity) to analyze 374 genes upregulated at D56 versus D10. A heat map presents the signals of microarray data of genes in one of the most upregulated network. (B) Downregulated networks were obtained using IPA to analyze 431 genes downregulated at D56 versus D10. A heat map presents the signals of rough microarray data of genes in one of the most downregulated networks. (C) The “Lipid Metabolism, Molecular Transport, and Small Molecule Biochemistry” is one of the most significant networks upregulated at D56 versus D10; it is presented with a graph made by IPA from the data of mice reared under CONT conditions. The values recorded under each gene represent the fold change of microarray gene expression data at D56 versus D10. IPA, ingenuity pathway analysis.

**Figure 4:**
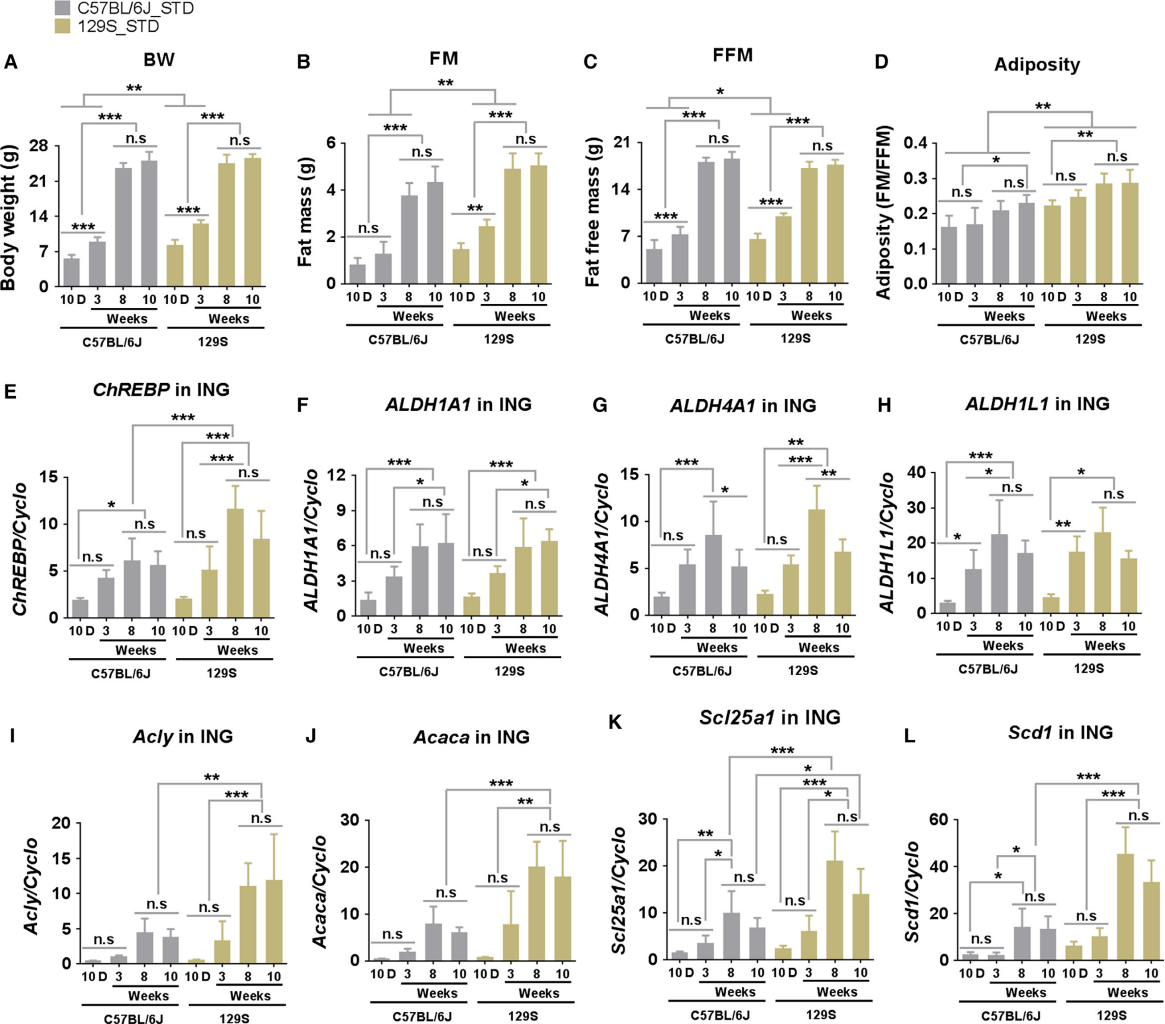
Developmental patterns of the mRNA expression for genes linked to lipid metabolism in B6 and 129 mouse strains fed a standard diet. Body weight (A), fat mass (B), fat‐free mass (C), and adiposity (D) of male B6 and 129S mice fed a STD were measured at 10 days (10D), 3, 8, and 10 weeks‐of‐age. mRNA levels (in 60 ng total RNA) of important genes involved in the network of “Lipid Metabolism, Molecular Transport, and Small Molecule Biochemistry” (Fig. 3) including the transcriptional factor *CheREBP* (E), the *ALD family (ALDH1A1, ALDH4A1*, ALDH1L1) (F–H) and lipogenic genes (*Acly*,* Acaca*,* Scl25a1*, and *Scd1)* (I–L) in inguinal fat of these mice were determined at indicated time points by qRT‐PCR. Data shown in graphs are the mean ± SD (error bars) with *n* = 7–10 (A–D) and 5–7 (E–L). Differences between groups were analyzed for statistical significance by two‐way ANOVA with multiple comparisons, significance levels indicated by *P ≤ 0.05, **P ≤ 0.01, and ***P ≤ 0.001.

The Venn analysis selected 374 genes that were significantly upregulated between D10 and D56 in inguinal fat of B6 mice raised under the three nutritional conditions and 431 genes that were downregulated (Fig. 3A and B). IPA identified 18 networks that were upregulated in 56‐day‐old fat versus 10‐day‐old fat (Fig. 3A) and 22 networks that were significantly represented in the downregulated genes (Fig. 3B). Among the 12 networks shown for each set, we selected the “Lipid Metabolism, Molecular Transport, and Small Molecule Biochemistry” network for further analysis (Fig. 3A and C). The microarray signal of each gene in this network is presented as a heat map (Fig. 3A). The heat map underscores the existence of patterns of expression determined by developmental age, compared to very modest effects of diet, even under the severe conditions of undernutrition in which dams were fed only 50% of the food consumed by control dams (See Methods and Material). The developmental genetic program of the adipocyte has a delayed activation of the lipogenic pathway in inguinal fat until early adulthood, that is, 56 days of age, which coincides with the onset of DIO in male B6 mice. Pathway analysis of the “Lipid Metabolism, Molecular Transport, and Small Molecule Biochemistry” network by IPA showed interactions between the ALD genes and lipogenic genes, and that their activities may be under the control of the transcriptional factor carbohydrate‐responsive element‐binding protein (ChREBP, also known as MLX‐interacting protein‐like, MLXIPL) (Fig. 3C). Previously, Kiefer et al. (2012) showed that ADLH1A1 enhanced fat accumulation by inhibiting the thermogenic program in white fat in mice. Since brown adipocytes are induced in inguinal fat by a developmental program between birth and 21 days of age (Chabowska‐Kita et al. 2015), the expression pattern of brown fat is consistent with low expression of *ALDH1A1* during the preweaning period and maximal expression at 8 weeks‐of‐age. In addition, genes with regulatory activity in the adipogenesis program, including *ChREBP*, and the genes, *ALDH1A1*,* ALDH4A1*, and *ALDH1L1*, and several genes of lipogenesis, including *Acly*,* Acaca*,* Scl25a1*,* Scd1*,* and Fasn*, reflected the development of an increased capacity for fat accumulation (Fig. 3A and C). We determined whether the developmental expression of these genes associated with lipogenesis had achieved a higher expression in B6 compared to 129S mice at 70 days of age, that is, when B6 mice were more susceptible to DIO (Fig. 4). Expression of these genes increased steadily from the postnatal period to 8 weeks‐of‐age (Fig. 4E–L) in parallel with the increase of body weight and fat mass (Fig. 4A and B). Correlation analysis between gene expression and adiposity in the experiment of Figure 4 ranged from low *R* ^*2*^ = 0.15; *P* = 0.04 for *Scl25a1* to high *R* ^*2*^ = 0.38; *P* = 0.0005 for *Scd1*. While the ALDH genes showed similar levels of expression in B6 and 129 mice (Fig. 4F–H), the levels of expression of ChREBP and the lipogenic genes were uniformly higher in 129 than in B6 mice (Fig. 4E, and I–L). This result is consistent with the contribution of lipogenic genes and *ChREBP* transcriptional factor in determining a higher adiposity and fat mass in 129 mice than in B6 mice when fed a low fat‐chow diet up to 8 weeks‐of‐age (Figs. 1 and 4). It may explain the higher sensitivity to adiposity of B6 mice fed a HFD beginning at 8 weeks‐of‐age, but this needs further investigation.

Additionally, Ingenuity Pathway Analysis of the 431 genes downregulated between D10 and D56 has revealed 22 significant gene networks. The function of these networks is largely associated with developmental processes and DNA metabolism, networks that you would expect to be upregulated during early development. None were linked directly to lipid metabolism (Fig. 3B).

### QTLs associated with genetic obesity in 129S and B6 mice provide candidate metabolic pathways to test for perturbations that affect diet‐induced obesity

An additional source of information to identify genes controlling the induction of obesity in 8‐week‐old B6 mice and not 129S mice may be found in the QTL studies carried out by Reed and colleagues (18–20). These QTL studies of obesity in a F2 cross between B6 and 129S mice were based on adiposity traits in mice between 6.8 and 12.6 months of age (Reed et al. 2003, 2006; Lin et al. 2013). DNA was genotyped with PCR‐based microsatellite markers and linkage was found to regions carrying several genes encoding enzymes of lipogenesis (Table 1). Intriguingly, candidate genes identified by the microarray analysis as described above in Figure 3 were also represented in Reed's QTLs, suggesting a potential involvement of these candidate genes in differential susceptibility to diet‐induced obesity. In addition to these genes of lipogenesis, identified from the QTL study, we selected mesoderm‐specific transcript (MEST) and secreted frizzled‐related protein 5 (SFRP5) for further analysis, since these genes have among the highest levels of induction of any known gene in adipose tissue in association with the increased adiposity in adult male B6 mice fed a high fat diet (Nikonova et al. 2008). In summarizing the adipogenic gene expression data for DIO during development, it is noteworthy that three independent studies point to the involvement of the genes we have focused on.

### Developmental sensitivity to DIO in B6 and 129S mice and its association with genes of adipose tissue expansion

**Figure 5:**
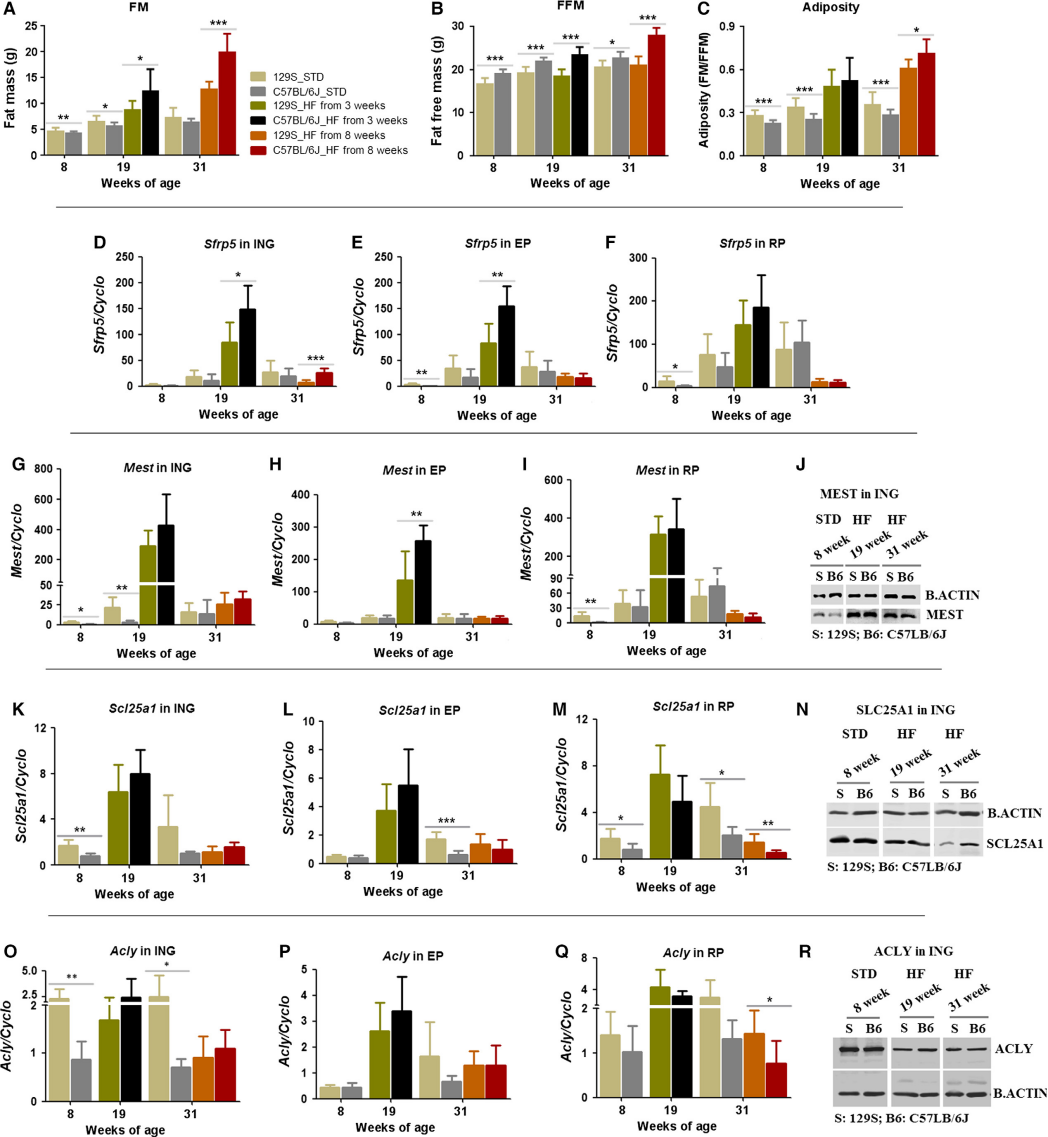
Developmental and nutritional effects on candidate gene expression between C57BL/6J and 129S mice. C57BL/6J and 129S males were fed with a high fat diet (HF) or a standard diet (STD) from 3 or 8 weeks‐of‐age. Fat mass (A), fat‐free mass (B), adiposity (C), and mRNA levels (in 60 ng total RNA) of selected candidate genes including *Sfrp5* (D–F), *Mest* (G–I), *Scl25a1* (K–M), and *Acly* (O–Q) were measured by qRT‐PCR in three white fat depots (ING, EP, and RP) at 8, 19, and 31 weeks‐of‐age. Mesoderm‐specific transcript, SCL25A1, and ACLY protein (J, N, and R) in ING fats were analyzed by western blot at indicated time points. Data shown in graphs are the mean ± SD (error bars) with *n* = 7–13. Significant levels are indicated by *C57BL/6J group relative to 129S group in the same diet. Differences between groups were analyzed for statistical significance by Student's *t*‐test with significance levels indicated by *P ≤ 0.05, **P ≤ 0.01, and ***P ≤ 0.001.

Until they are 8 weeks‐of‐age, both B6 and 129S mice are resistant to DIO (Figs. 1, 2 and 4D); however, compared to 129S, B6 mice show an enhanced increase in adiposity at 19 and 31 weeks‐of‐age (Fig. 5A–C). Consistent with the enhanced DIO in B6 mice, GTT and ITT indicated that B6 mice fed a high fat diet were glucose intolerant and had reduced insulin sensitivity at 30 weeks‐of‐age compared to 129 mice (Fig. 2G and H). Since previous experiments showed that both MEST and SFRP5 were highly correlated with adiposity in adult mice (Nikonova et al. 2008), we expect to find similar correlations in this experiment. This expectation was observed for adiposity and gene expression before 8 weeks‐of‐age in that there was no increase in adiposity in mice fed a high fat diet, nor any corresponding induction of *Mest* and *Sfrp5*. A positive correlation was observed in both 129 and B6 mice at 19 weeks‐of‐age for *Sfrp5* and *Mest* expression in epididymal fat fed a high fat diet, but not in inguinal or retroperitoneal fat depots. 129 mice also showed significantly elevated expression of *Sfrp5* and *Mest*. Unexpectedly, even though adiposity continued to increase between 19 and 31 weeks‐of‐age, gene expression in mice 31 weeks‐of‐age was suppressed at the mRNA level for *Sfrp5* (Fig. 5D–F) and for *Mest* (Fig. 5G–I) and also at the protein level though not as strongly as for mRNA (Fig. 5J). At 31 weeks, the same degree of suppression was observed in ING, EP, and RP fat of mice fed a HF; however, higher levels of *Sfrp5* and *Mest* were measured in the RP of mice fed a low fat chow (Fig. 5F and I). Overall, expression of the *Mest* and *Sfrp5* genes has become uncoupled from the adiposity index of the tissue in aging mice, that is, high adiposity was not associated with high gene expression.

To investigate further the uncoupling of expression of genes of lipid metabolism from the conditions of adiposity, we analyzed expression of the lipogenic genes (Fig. 5K–R). *Scl25a1* (Fig. 5K–N) responded very much like *Mest* and *Sfrp5*, that is, low expression up to 8 weeks‐of‐age, high mRNA levels at 19 weeks‐of‐age, and then suppressed levels in 31‐week‐old mice despite high adiposity. *Acly* mRNA levels showed no consistent relationship to age or diet with increasing age (Fig. 5O–Q). The levels of protein for SLC25a1 and ACLY, as estimated by immunoblots (Fig. 5L and S) were strong and were reduced with increasing age. Although 129 mice showed significant increases in adiposity after 19 and 31 weeks on a high fat diet, nevertheless, the glucose and insulin tolerance was essentially normal when compared to that of B6 mice (Fig. 7B–E).

### Effects of age, diet, and genotypes on chromatin structure of *Sfrp5* and *Acly*

#### DNA methylation

**Figure 6:**
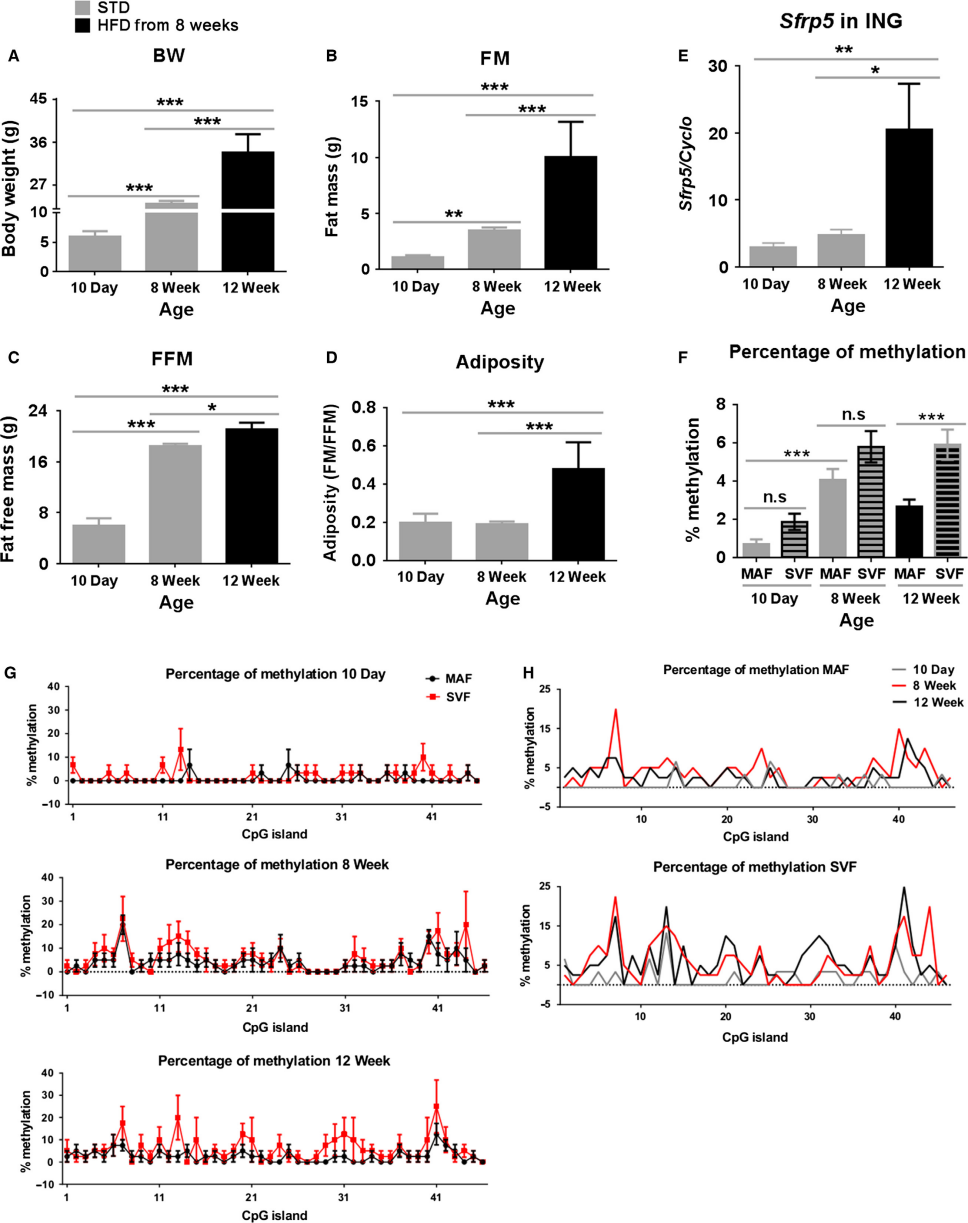
The effect of DNA methylation on the gene expression during development and under various diets. C57BL/6J males were fed with a STD from weaning to 8 weeks‐of‐age, and then one group was fed a high fat diet (HF) until 12 weeks‐of‐age (A–D). Body composition of mice measured at 10 days, 8 and 12 weeks‐of‐age. Data are the mean ± SD (error bars) with *n* = 15, 4, and 4 for 10 days, 8 weeks, and 12 weeks‐of‐age, respectively (E). Relative mRNA levels (in 60 ng total RNA) of *Sfrp5* were determined in whole ING fat tissues examined by real‐time PCR. Data are presented as the mean ± SEM (error bars) with *n* = 3, 4, and 4 at 10 days, 8 weeks, and 12 weeks‐of‐age, respectively. Three duplicates of individual groups are shown. In the 10‐day‐old group, each *n* contained a pool of ING tissue from five animals (F). Mean percentage values of methylation of the 46 CpG's islands in the promoter region of *Sfpr5* gene in the MAF and the stromal vascular fraction (SVF) from ING fat tissue. Data are the mean ± SEM (error bars) with *n* = 30, 40, and 40 clones for 10 days, 8 weeks, and 12 weeks‐of‐age, respectively (G). Comparison of methylation pattern of DNA isolated from SVF and MAF of ING fat collected from 10 day (*n* = 3), 8 week (*n* = 4), and 12‐week (*n* = 4)‐old mice (H). Sodium bisulfite sequencing analysis of DNA from cellular fractions (MAF, SVF) isolated from ING fat collected from animals at different ages, 10 days (*n* = 3), 8 weeks (*n* = 4), and 12 weeks (*n* = 4). Data are the means only. Differences between groups were analyzed for statistical significance by one‐way ANOVA with multiple comparisons, significance levels indicated by *P ≤ 0.05, **P ≤ 0.01, and ***P ≤ 0.001. MAF, mature adipocyte fraction; STD, standard chow diet; SVF, stromal vascular fraction.

The strong influence of age during early development and aging on gene expression prompted us to investigate whether differential DNA methylation of these genes of lipid metabolism occurs during development and under different diets. As a test gene, we selected the *Sfrp5* gene for analysis because it has clear, unambiguous patterns of expression, that is, it has virtually no expression from birth until 8 weeks‐of‐age, but is highly induced at 8 weeks‐of‐age when the mice are fed a high fat diet. If the expression of *Sfrp5* is controlled by DNA methylation, differences should be found in the neonatal mouse compared to the adult. We measured the percentage of methylated CpG islands in the *Sfrp5* promoter region of the MAF and the SVF isolated from ING fat of 10‐day‐old mice, 8‐week‐old mice fed a STD, and 12‐week‐old mice fed a HF (Fig. 6). In this experiment, *Sfrp5* mRNA levels were fourfold elevated in ING fat of 12‐week‐old mice fed a HF compared to 10 day and 8‐week‐old mice fed STD diet (Fig. 6E), which is consistent with HF‐induced increase of body composition (Fig. 6A–D). At the same age and with the same diet, there was no significant difference in *Sfrp5* methylation between SVF and MAF in mice at 10 days or 8 weeks‐of‐age (Fig. 6F). In contrast, cellular fractions of 12‐week‐old mice had a significantly lower percentage of *Sfrp5* methylation in MAF than SVF (Fig. 6F and G). The higher *Sfrp5* DNA methylation was found in both SVF and MAF of adult mice compared to 10‐day‐old mice, but there was no statistical difference in *Sfrp5* methylation between 8 and 12‐week‐old mice (Fig. 6F and H). DNA methylation of the promoter region of a specific gene should suppress that gene expression; however, it was apparent that the percentage of methylation in *Sfrp5* promoter was not connected to *Sfrp5* expression in ING fat depots in our model, for example, ING fat of 12‐week‐old‐mice eating HF had not only the highest *Sfrp5* mRNA but also high *Sfrp5* methylation (Fig. 6E and F). Thus, our data indicated that the variation in mRNA *Sfrp5* expression in inguinal adipose depots following development and under different diets was not controlled by changes in DNA methylation in *Sfrp5* promoter. This observation is consistent with the DNA methylation patterns of the *Mest* promoter (Koza et al. 2009).

#### Histone acetylation

**Figure 7:**
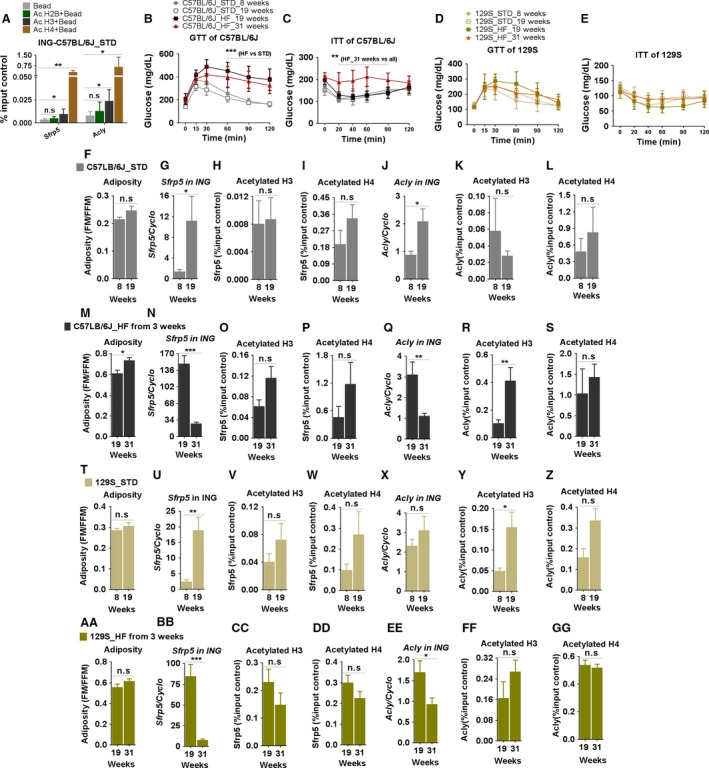
Developmental, genetic, and nutritional effects on histone acetylation at *Sfrp5* and *Acly* in C57BL/6J and 129S mice. C57BL/6J and 129S males were fed with a high fat diet (HF) or a STD from 3 or 8 weeks‐of‐age. Glucose and insulin sensitivity of C57BL/6J (B–C) and 129S mice (D–E) were determined at 8, 19, and 31 weeks‐of‐age. Histone 2B, 3, and 4 acetylation at Sfrp5 and Acly genes was determined by ChIP assay in ING of adult C57BL/6J mice fed a STD diet (A). Adiposity, mRNA levels (in 60 ng total RNA) of *Sfrp5* and *Acly*, acetylation of histone 3 and 4 associated with *Sfrp5* and *Acly* in ING fats from C57BL/6J on STD (F–L) and HF (M–S), and 129S on STD (T–Z) and HF (AA–GG) mice were determined at the indicated time points. Data shown in graphs are the mean ± SD (error bars) with *n* = 3–4 (A, F‐GG) and 6–11 (B, C, D, and E). Differences between groups were analyzed for statistical significance by Student's *t*‐test (A, F‐GG) or two‐way ANOVA (B, C, D, and E) with multiple comparisons, significance levels indicated by *P ≤ 0.05, **P ≤ 0.01, and ***P ≤ 0.001. STD, standard chow diet.

In the absence of significant changes in DNA methylation, we assessed whether modifications of histone–chromatin structure occurred with a chromatin immunoprecipitation (ChIP) assay. The adiposity and glucose metabolism as evidenced by GTT and ITT assays showed that B6 mice were glucose and insulin intolerant only when fed a high fat diet, whereas 129S mice were normal (Fig. 7B–E). Adiposity was induced in B6 mice fed a HF, but not a STD diet (Fig. 7F and M). Adiposity was neither induced in 129S mice fed a HF (Fig. 7AA), nor was there evidence of glucose intolerance (Fig. 7D), although the experiment reported 129S mice showed an unexpectedly high adiposity (Fig. 7T and AA) compared to adiposities for 129 mice in Fig. 1F; however, it was not accompanied by an abnormal GTT (Fig. 7D). An additional complication is that the levels of mRNA for *Sfrp5* and *Acly* are reduced in the expression in aging tissue, that is, 31 weeks, despite the increase in adiposity (Fig. 7N and Q; BB and EE). The acetylated status of three important histones (H2B, H3, and H4) associated with active gene expression (Wellen et al. 2009) was tested in inguinal fat tissues of B6 and 129 mice fed STD until 8 weeks‐of‐age prior to the onset of DIO (Fig. 7F–L and T–Z) and in B6 and 129 mice fed STD until 8 or 19 weeks‐of‐age, and then HF until 19 or 31 weeks‐of‐age (Fig. 7M–S and AA–GG). At binding sites in the promoters of *Sfrp5* and *Acly* genes (Linhart et al. 2007; Neess et al. 2011), we found enrichments for binding of H3 and H4 acetylation, but not H2B acetylation (Fig. 7A). We therefore tested for binding of acetylated H3 and H4 in B6 and 129S mice fed either STD or HF diets at different ages. Binding of the acetylated histones to *Sfrp5* and *Acly* was very low in young B6 and 129 mice, then subsequently increased in mice older than 8 weeks‐of‐age; however, the only significant difference between young and older mice was found for acetylated histone H3 (Fig. 7R and Y). We were not able to determine whether the genetic background, B6 versus 129, the diet, or obese states are associated with differences in acetylated histones for the *Sfrp5* or *Acly* genes in inguinal fat.

## Discussion

Since the initial studies by Surwit and colleagues on the enhanced sensitivity of B6 mice to develop obesity when fed a high fat diet, many studies have elaborated on the obese phenotype in the B6 mice (Surwit et al. 1988). Consequently, diet‐induced obesity in the male B6 may be one of the best experimental models for multifactorial obesity in humans. This study to identify pathways controlling diet‐induced obesity is based on the selection of metabolically functional candidate genes, identified by microarray analysis, that were further tested to identify the interaction of genotype, diet, and developmental age on the expression of candidate genes of adipose tissue expansion, as well as assessing epigenetic mechanisms based upon DNA methylation and histone acetylation of a well‐described marker gene of DIO. We found that expression patterns of *Sfrp5* correlated with obesity phenotypes, but the epigenetic mechanisms we tested did not.

Four key features of DIO in the B6 mouse have informed the design of our study: (1) the acquisition of an enhanced capacity for lipid accumulation is delayed until 8 weeks‐of‐age. This capacity correlates with the ability of a set of adipose tissue expansion genes, including *Mest*,* Sfrp5*, and *Bmp3* (Koza et al. 2006; Voigt et al. 2015), to be induced in response to a positive energy balance, not a high fat diet. Mice fed a HF when housed in the cold (4°) have a high energy expenditure that prevents the development of a positive energy balance. Accordingly, the mice are not obese and they do not induce gene expression (Nikonova et al. 2008; Jura et al. 2015). (2) In addition, we show that the induction of gene expression in response to increased adiposity does not occur in the neonatal mouse, that is, high levels of *Mest* expression in the neonatal mouse are not associated with excess fat accumulation (Nikonova et al. 2008; Chu et al. 2014) and this indicates that conditions at different stages of development will determine whether the *Mest* gene will drive the expansion of adipose tissue. (3) There is a subtle involvement of the state of energy balance during the weaning period on long‐term susceptibility to DIO (Koza et al. 2006; Chabowska‐Kita et al. 2015). DIO is reduced in adult mice when they were undernourished during the gestation period, and DIO is also reduced in adult mice that experienced increased energy expenditure from cold‐activated thermogenesis during early postnatal development. Under conditions of undernutrition, mice are in negative energy balance, whereas during a cold challenge, which creates a state of increased energy expenditure, the pups increase energy intake to maintain energy balance. This suggests that it is not the state of energy balance per se that affects long‐term susceptibility to obesity. Rather, it is cellular responses to maintain energy balance whether from caloric deficiency or increased caloric expenditure to maintain body temperature. Therefore, the states of energy metabolism in both experiments led to reduced adiposity and blood glucose in the preweaning mice and suppressed DIO in adult mice (Koza et al. 2006; Chabowska‐Kita et al. 2015). (4) The sensitivity to DIO in B6 offspring, raised under standard dietary conditions, does not occur until mice are adults, that is, 8 weeks‐of‐age, as shown by Bachmanov et al. (2001). This underscores the idea that DIO in B6 male mice is basically a developmental problem.

A key aspect of the B6/129S model is to assess how the adiposity phenotypes and the candidate metabolic genes are affected by developmental age. Bachmanov et al. (2001) clearly showed that the induction of obesity by a high fat diet, but not other macronutrients, in B6 mice, but not 129S mice, did not occur until after 8 weeks‐of‐age. This phenotype was replicated at Pennington Biomedical Research Center in Baton Rouge, LA in Figure 1 (this paper) and in Poland (Fig. 2) together with an assessment of the effects of the maternal environment with a cross‐fostering design (Fig. 1). Cross‐fostering was an important experiment because of the known effects of maternal behavior and epigenetics on development (Weaver et al. 2004; Kozak et al. 2010). This experiment shows that the maternal postnatal environment did not affect the development of obesity; rather, the adiposity phenotype is dependent on the respective genetic background. However, evidence is emerging from other studies that the genetic constitution interacting with the nutritional environment during the postnatal period may be especially important to the long‐term development of obesity (Koza et al. 2006; Kozak et al. 2010; Lasar et al. 2013).

Changes with age were not only evident during early development, but also in aging mice, that is, in 31‐week‐old mice. In these mice fed a high fat diet, adiposity continued to increase, but the adipose tissue biomarkers almost uniformly, with the exception of *Acaca (data not shown)*, had significant reductions in expression. The suppression of gene expression uncoupled to the level of adiposity with aging was also observed recently in a very striking manner in *ob/ob* mice at a time that coincided with the attainment of maximal adiposity (Jura et al. 2015). These studies underscore the plasticity or instability of gene expression in adipose tissue. We do not know what causes the suppression of gene expression in the aging tissue. From this study, the methylated state of the gene was not changing and although the binding of acetylated histones to *Sfrp5* and *Acly* was detected at 31 weeks‐of‐age, this in itself was not sufficient for active gene expression.

Age‐dependent differences in systems associated with lipid metabolism, such as the brite cell phenotype, occur in B6 mice. Brite cell induction occurs at thermoneutrality (29°C) between birth and weaning, but no induction is detectable during the postweaning period until the ambient temperature is below 22°C (Chabowska‐Kita et al. 2015). Presumably, the reduced induction of brite cells in adult mice should render these mice more sensitive to obesity, which in fact is observed (Guerra et al. 1998). Lasar et al. (2013) have proposed that the higher adiposity in 129S preweaning mice is due to higher brite cell expression. Our results comparing the development of inguinal fat and iBAT in B6 and AxB8 mice do not agree with their observations (Chabowska‐Kita et al. 2015). Despite higher levels of brite cell expression in AxB8 mice, adiposity in these mice during the lactation period is not less (Chabowska‐Kita et al. 2015). In addition, Almind et al. (2007) have argued for a role for intermuscular *Ucp1* expression in the reduced adiposity in adult 129 mice. However, since the level of *Ucp1* expression is so extraordinarily low in skeletal muscle, brite cell expression is not likely to have an impact on diet‐induced obesity in B6 mice.

In the B6 model, Kiefer et al. (2012) found that retinaldehyde dehydrogenase 1 (*ALDH1A1*) inhibits formation and activity of brown fat cells in white fat depots, therefore, its expression could enhance fat accumulation. This conclusion is confirmed again by our work, among the lipid metabolism and development networks determining the induction of fat accumulation at 8 weeks‐of‐age, we found that the expression of *ALDH1A1* and other ALD genes correlated with the lipogenic genes, ChREBP and increases in adiposity. The data indicate that the striking increase of fat mass in 8‐week‐old mice was accompanied by higher mRNA expression of *ALDH1A1*,* ALDH4A1*,* ALDH1L1*,* Acly*,* Acaca*,* Scl25a1*,* Scd1*, and *ChREBP* in the inguinal fat.

In summary, our experiments suggest that the variation in DIO between B6 and 129S mice is not only a reflection of differences in genetics and nutrition, but also this variability is highly dependent on development age. We propose that an explanation for some of the DIO variability in this obesity model is that developmental phases affect the expression and function of important adipose biofunctional markers such as *Mest*,* Sfrp5*,* Scl25a1*, and *Scd1* in white fat depots. We also found that the developmental susceptibility of B6 mice to DIO at 8 weeks‐of‐age is accompanied by changes in expression of some adipose functional markers and accordingly may involve genes functioning in networks of lipid metabolism such as ALD and lipogenesis genes and the transcriptional factor ChREBP. Although unequivocal evidence for changes in chromatin structure associated with DNA methylation or histone acetylation was not obtained, the patterns of gene expression of pathways of lipid metabolism suggest that further experimentation on the role these pathways may play in susceptibility to obesity is warranted.

## Conflict of Interest

None declared.
